# Serial evaluation of swallowing function in a long-term survivor of V180I genetic Creutzfeldt-Jakob disease

**DOI:** 10.1080/19336896.2020.1787090

**Published:** 2020-07-05

**Authors:** Kenjiro Kunieda, Yuichi Hayashi, Megumi Yamada, Masahiro Waza, Tomonori Yaguchi, Ichiro Fujishima, Takayoshi Shimohata

**Affiliations:** a Department of Neurology, Gifu University Graduate School of Medicine, Gifu, Japan; b Department of Rehabilitation, Hamamatsu City Rehabilitation Hospital, Shizuoka, Japan; c Department of Neurology, Kakamigahara Rehabilitation Hospital, Kakamigahara, Japan

**Keywords:** Genetic Creutzfeldt-Jakob disease, V180I mutation, long-term survivor, dysphagia, pseudobulbar palsy

## Abstract

Swallowing function in long-term survivors with Creutzfeldt-Jakob disease (CJD) remains unknown. Herein, we demonstrated serial evaluation of swallowing function in a case with V180I genetic CJD (gCJD) using videofluoroscopic examination of swallowing (VF). A 69-year-old woman was admitted to our hospital because of bradykinesia and memory disturbances 4 months after the onset of symptoms. Neurological examination revealed dementia, bradykinesia and frontal signs. Diffusion-weighted MRI revealed bilateral cortical hyperintensity in the frontal, temporal, and parietal cortices, and *PRNP* gene analysis indicated a V180I mutation. Her dysphagia gradually progressed, and she received percutaneous gastrostomy 42 months after the onset. VF was performed at 27, 31, 39, and 79 months after the onset. Although bolus transport from oral cavity to pharynx gradually worsened and initiation of the pharyngeal swallow was gradually delayed, the pharyngeal swallowing function was preserved even at 72 months after onset. MRI revealed no apparent atrophy of brainstem, and single photon emission computed tomography showed preserved regional cerebral blood flow in the brainstem. These findings suggest that the pathophysiology of dysphagia in a long-term survivor of V180I gCJD is that of pseudobulbar palsy, likely owing to preserved brainstem function even in the akinetic mutism state.

## Introduction

Genetic Creutzfeldt-Jakob disease (gCJD) with a V180I mutation in the *PRNP* gene is the most common type of gCJD in Japan, and accounts for 41% of gCJD patients [1]. However, V180I gCJD is rare in European countries and the United States [2,3]. Its clinical characteristics are unique: elderly-onset, slow progressive course, sporadic fashion, and cortical oedematous hyperintensity on diffusion-weighted magnetic resonance imaging (DW-MRI) [4,5]. The average disease duration in V180I gCJD is 23–27 months [5]; however, long-term survivors (more than 5 years) have also been reported [6,7]. Appropriate care for dysphagia, a symptom of gCJD, is considered a factor that enables long-term survival of these patients [6]. However, the swallowing function of a long-term survivor of V180I gCJD patient remains unknown. In addition, there are no reports on how long oral intake could be continued in the patients. Herein, we report the serial evaluation of swallowing function of a long-term survivor of V180I gCJD using videofluoroscopic examination of swallowing (VF).

## Patient

**Figure 1.**
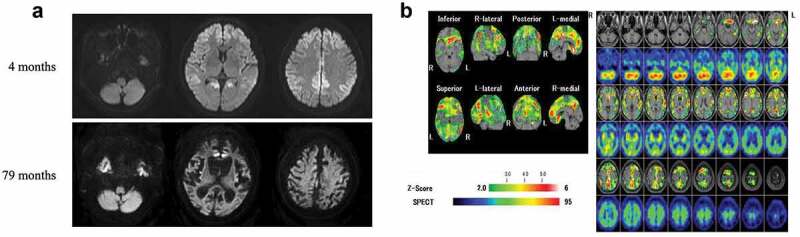
Magnetic resonance imaging (MRI) and single photon emission computed tomography (SPECT) of the patient. (a) Images from follow-up diffusion-weighted MRI 79 months after the onset revealed that atrophy of the brainstem was not apparent. However, the severe cerebrum atrophy was found. (b) SPECT images at 79 months after the onset revealed decreased regional cerebral blood flow (rCBF) in the bilateral frontal and parieto-temporal lobes; however, rCBF was preserved in the brainstem and cerebellum. The eZIS analysis of ^99mTc^-ECD SPECT images revealed decreased rCBF. A higher Z-score indicates a lower rCBF. The Z-score of 2 to 6 is indicated by the green to red (lower rCBF) colour gradient.

A 69-year-old woman was admitted to our hospital after 4 months of experiencing bradykinesia and memory disturbances without any family history. Neurological examinations confirmed a diagnosis of dementia (27/30 points on the Mini-Mental State Examination [MMSE] at 4 months after the onset of symptoms, which had deteriorated to 2/30 points on the MMSE at 9 months after the onset), and revealed bradykinesia and frontal signs, as described previously [8]. Startled responses and forced laughing were observed starting from 5 and 8 months after onset, respectively. However, forced crying was not observed. *PRNP* gene analysis revealed a V180I mutation with methionine/valine heterozygosity at codon 129. Brain DW-MRI showed bilateral cortical hyperintensity in the frontal, temporal, and parietal cortices (Figure 1(a)). Thereafter, ^99m^Tc-ethylcysteinate dimer single photon emission computed tomography (^99m^Tc-ECD-SPECT) images revealed decreased regional cerebral blood flow (rCBF) in the frontal and parietal lobes, bilaterally. Periodic sharp wave complexes were not observed on the electroencephalogram [8].

Her condition slowly deteriorated to a bedbound state by one year after the onset of symptoms; however, she was fed orally by a caregiver. She exhibited dysphagia classified as level 8 via the Food Intake LEVEL Scale (FILS) (the patient eats three meals by excluding food that is particularly difficult to swallow) [9]. Her condition slowly deteriorated to a state of akinetic mutism. Initial VF was performed at 27 months after the onset. As test foods, jelly, natural liquid, and chopped foods were used to evaluate the swallowing function in the sitting position. Although bolus transport from the oral cavity to the pharynx was slightly poor, delayed swallowing reflex was not observed. Follow-up VF at 31 and 39 months after symptom onset revealed that bolus transport from the oral cavity to the pharynx was slightly worse than in the previous VF. Initiation of pharyngeal swallow was delayed and laryngeal elevation delayed time (LEDT) [10] was 3.67 s. At 38 months after the onset of symptoms, a videoendoscopic examination of swallowing (VE) revealed pharyngeal myoclonus and no vocal cord paralysis.

As the patient gradually took longer to intake food orally with assistance and the burden of caregiver also increased, she received percutaneous gastrostomy 42 months after the onset. Her family eagerly wanted her to eat by her mouth and she was fed orally by a caregiver for as long as possible. At that time, her ability to swallow was estimated to be level 5 (easy-to-swallow food orally ingested in one to two meals, but alternative nutrition is also given) as per FILS.

**Figure 2.**
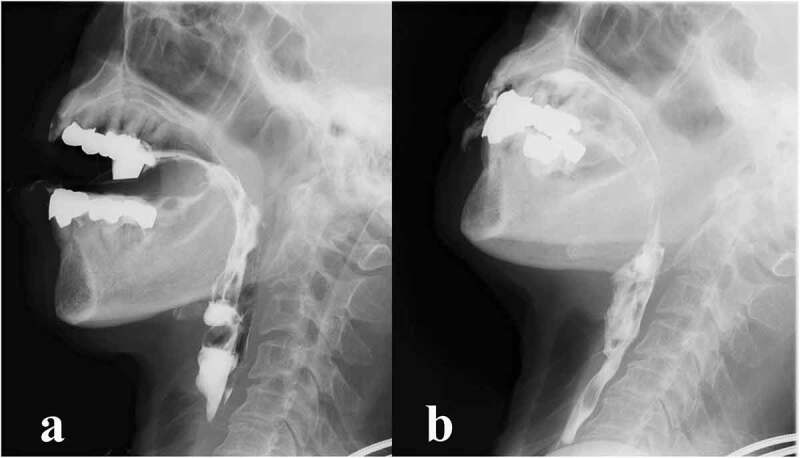
Videofluoroscopic examination of swallowing (VF) of the patient.

VF was performed at 79 months after the onset (Figure 2). Although bolus transport from the oral cavity to the pharynx had worsened and bolus in the oral cavity spilled out of the mouth, adjustment of positioning such as reclining posture improved bolus transport to the pharynx. Furthermore, the initiation of the pharyngeal swallow was even more delayed, although the swallowing function was preserved without pharyngeal residues and aspiration. At that time, brain MRI showed progressive cerebral atrophy (Figure 1(a)), but brainstem and cerebellar atrophy was not apparent. ^99m^Tc-ECD-SPECT images at 79 months revealed severely decreased rCBF in the frontal and parieto-temporal lobes bilaterally; however, rCBF was preserved in the brainstem and cerebellum (Figure 1(b)). At the age of 75, the patient was still alive and continued to intake food orally with assistance.

VF of the patient in lateral view at 79 months after the onset of symptoms. Fluoroscopic images (a) before swallowing reflex and (b) and during swallowing. (a) Bolus transport from the oral cavity to the pharynx was poor and initiation of the pharyngeal swallow was delayed. (b) Once the swallowing reflex was triggered, the bolus passed through the penetration pharynx into the upper oesophagus without laryngeal or aspiration

## Discussion

To best our knowledge, this is the first report to evaluate serial swallowing function using VF in a long-term survivor of V180I gCJD. We demonstrated that swallowing function, especially brainstem swallowing reflex, was well preserved even in an akinetic state of V180I gCJD.

According to our serial VF studies, the swallowing pattern was that of pseudobulbar palsy. Bolus transport from the oral cavity to the pharynx was poor and initiation of the pharyngeal swallow was delayed. The delayed LEDT also reflected pathophysiology of the delayed onset of swallowing reflex. Meanwhile, the pharyngeal swallowing function was preserved without pharyngeal residue and aspiration. VF findings in the present patient are typical to those of patients with pseudobulbar palsy due to bilateral cortico-bulbar tract impairment [10]. Moreover, serial MRI and SPECT findings supported the diagnosis of pseudobulbar palsy [6].

This consideration is consistent with previous studies showing that the brainstem function was preserved in V180I gCJD patients. Serial SPECT studies indicate preserved rCBF in the brainstem and cerebellum [8]. According to previous reports of autopsy-verified V180I gCJD patients, while diffuse spongiform changes were observed in the cerebral cortex, cerebellar and brainstem lesions were absent or mild despite the prolonged duration of the disease [5–7,11,12]. The absence of vocal cord paralysis in VE of this patient also supported preserved brainstem function. Findings from these studies and reports showed preservation of brainstem function in V180I gCJD patients. The central pattern generators (CPGs) for the pharyngeal and oesophageal phases of swallowing are located in the nucleus tractus solitarius [13]. In cases where brainstem neurological function is preserved, the pattern of swallowing might also be preserved, as in the current patient, because CPGs are not impaired.

To better understand dysphagia in V180I gCJD patients, we reviewed articles that described nutritional administration methods for ten patients including the current patient (Table 1) [6,8,12,14–17]. Six of the ten patients (60%) could orally intake food after deteriorating to the state of akinetic mutism. The average duration of oral intake in these patients after reaching to the state of akinetic mutism was 16.6 ± 3.3 months. Two patients in particular, including the current patient, had longer oral intake durations of more than five years. Although a patient with V180I gCJD reaches a state of akinetic mutism, we emphasize that clinicians should consider evaluating swallowing function with VE and/or VF. With regard to the pathophysiology of dysphagia, these previous reports revealed that the brainstem was preserved and that these findings supported the diagnosis of pseudobulbar palsy. Some V180I gCJD patients could continue oral intake until death, as well as those with MM2-cortical type sporadic Creutzfeldt-Jakob disease [18]. In the present patient, her family had a desire that she ingests orally. Continuing oral intake might be meaningful for families and caregivers with appropriate care. Assessment of the swallowing function and appropriate swallowing techniques, such as dietary modification and postural adjustment, may improve swallowing and avoid aspiration.

**Table 1.** Clinical course of patients with V180I genetic Creutzfeldt-Jakob disease except for patients with V180I/M232R combined mutation.

Case No.	Author (y)	Onset of Age (yrs)	sex	Polymorphism at the codon 129 in the *PRNP gene*	Oral intake or Tube-feeding (Duration between onset and starting tube-fed) (m)	Duration of oral intake after onset (m)	Duration between onset and akinetic mutism (m)	Duration of oral intake after deteriorating akinetic mutism (m)	Cause of death (Duration between onset and death) (m)	MRI findings	Autopsy finding of brainstem	Evaluation of brainstem function	Evaluation of swallowing function
1	Iwasaki (2019) [14]	87	F	M/M	Oral intake	22	18	4	Alive	Cerebral hyperintensity Brainstem preserved	-	N.D.	N.D.
2	Iwasaki (2018) [15]	86	F	M/M	Oral intake	10	-	N.D.	General weakness (10)	Cerebral hyperintensity	No apparent atrophy	N.D.	N.D.
3	Iwasaki (2017) [16]	78	F	M/M	Gastrostomy (30)	30	16	14	Respiratory failure (33)	Cerebral hyperintensity	N.D.	N.D.	N.D.
4	Iwasaki (2017) [16]	76	F	M/M	Gastrostomy (38)	34	17	17	Alive >77 months	Cerebral hyperintensity	-	N.D.	N.D.
5	Iwasaki (2017) [16]	69	F	M/M	Gastrostomy (14)	14	13	1	Alive >49 months	Cerebral hyperintensity	-	N.D.	N.D.
6	Hayashi (2016, 2020) [6,8]	78	F	M/M	Tube-fed (47)	14	14	0	Pneumonia (61)	Cerebral hyperintensity Cerebral atrophy Brainstem preserved	No apparent atrophy	N.D.	N.D.
7	Hayashi (2016) [8]	74	F	M/V	Tube-fed (20)	20	20	0	Alive	Cerebral hyperintensity Cerebral atrophy Brainstem preserved	-	N.D.	N.D.
8	Deguchi (2012) [17]	79	F	N.D.	Oral intake	60	18	42	Alive	Cerebral hyperintensity Cerebral atrophy Brainstem preserved	-	N.D.	N.D.
9	Iwasaki (2011) [12]	73	F	M/M	Tube-fed	22	22	0	Respiratory failure (102)	Cerebral hyperintensity Cerebral atrophy Brainstem preserved	No apparent atrophy	N.D.	N.D.
10	Current patient	69	F	M/M	Gastrostomy (42)	83	12	71	Alive	Cerebral hyperintensity Cerebral atrophy Brainstem preserved	-	VE	VE, VF
	AVG ± SD (range)	77.0 ± 5.8 (69–87)	F (10/10)		29.3 ± 13.6 (14–47) (n = 6)	30.9 ± 23.2 (10–83) (n = 10)	16.6 ± 3.3 (12–22) (n = 9)	16.6 ± 24.6 (0–71) (n = 9)					

y: year, FILS: Food Intake LEVEL Scale, F: female, N.D.: not described, VE: videoendoscopic examination of swallowing, VF: videofluoroscopic examination of swallowing, AVG: average, SD: standard deviation

This study has several limitations. First, pathological analysis was not performed since the patient remained alive. Therefore, further studies on the neuropathological findings of this patient are needed. Second, this was a single case report; hence, future work and evaluation of additional cases are necessary.

## Conclusion

Swallowing function of patients with V180I gCJD might be preserved even in the akinetic mutism state. The pathophysiology of dysphagia is considered to show a pseudobulbar palsy pattern.
